# Social values, self- and collective efficacy explaining behaviours in coping with Covid-19: Self-interested consumption and physical distancing in the first 10 days of confinement in Spain

**DOI:** 10.1371/journal.pone.0238682

**Published:** 2020-09-17

**Authors:** Carmen Tabernero, Rosario Castillo-Mayén, Bárbara Luque, Esther Cuadrado

**Affiliations:** 1 Instituto de Neurociencias de Castilla y León (INCYL), University of Salamanca, Salamanca, Spain; 2 Faculty of Educational Sciences and Psychology, University of Córdoba, Córdoba, Spain; Middlesex University, UNITED KINGDOM

## Abstract

The appearance of a new coronavirus (Covid-19) and its rapid expansion throughout the world has forced all countries to establish regulations based on social confinement. In the early days of a pandemic, the adherence to regulations is crucial to be able to block its spread. This research aims to analyse the relationship between motivational variables associated with physical distancing and self-interested consumption behaviours in the first 10 days of confinement in Spain. A total of 1,324 people participated throughout the country (mean age 28.92 years). Participants answered an online survey about socio-demographic, motivational variables, which included a) risk information seeking, b) confidence in self- and collective efficacy in coping with the pandemic, and c) the four higher-order personal values ‒conservation (security, conformity, and tradition), self-transcendence (universalism and benevolence), openness (self-direction actions and stimulation), and self-improvement (hedonism and power) ‒ and the aforementioned behaviours in coping with Covid-19. Results showed a positive association between self- and collective efficacy and both coping behaviours analysed: a protective role of conservation values on normative behaviours; and a negative relationship between self-transcendence values and self-interested consumption. Additionally, risk information seeking was positively associated with the development of physical distancing behaviour.

## Introduction

The world is becoming embroiled in a global threat from the spread of a new coronavirus identified as SARS-CoV-2 after its appearance in Wuhan (China) in December 2019 and its rapid spread to all six continents, leading to the World Health Organization declaring the situation to be a global pandemic on March 11, 2020 [1]. By then the virus had already spread, with Italy being the first affected European country, and in fact the Italian government had declared a state of emergency on January 31 [2]. Weeks later, on March 14, the Spanish government declared a state of alarm throughout the country, while other countries were simultaneously signing up to the regulations to establish isolation enforcement of their entire populations [3]. Strict adherence to the rules of social isolation is extremely important from day one to stop the spread of the virus. This new global situation requires–perhaps more than ever–a collective response that will therefore become a global message for everyone (“Let’s stop this virus together” or “Coronavirus is a weak enemy if we fight it together”). However, partially, the responsibility for slowing down the spread of the virus rests with the sum of individual behaviours, and for this reason, governments proclaim individual responsibility (“If you protect yourself, you protect everyone”) and individual efforts to self-isolate (“Stay at home to stay safe”). Furthermore, by being aware of the need to provoke collective action, government messages exalt national social values as “a collective national effort”, creating the perception of unity by asking for trust in others (“We´ll stop it when you trust that we’re going to get through this”). These messages try to induce in individuals both a belief in their ability to stop the virus with their individual actions and a belief that if people follow the behavioural rules, we will be able to stop the virus together. Individuals’ beliefs in their capabilities to mobilize the motivation, cognitive resources, and courses of action needed to exercise control over given events correspond with the construct of self-efficacy beliefs proposed by Bandura [4]. Self-efficacy judgments influence the types of individual actions chosen to deal with potential threats that may occur in a global pandemic and perseverance in the face of adversity.

Alarming situations generate in individuals a conflict derived from a social dilemma, on the one hand, in the face of the perception of scarce resources (such as food or medicines), as well as individualistic behaviour–perhaps selfish consumption behaviour or acting in one’s short-term self-interest–and self-protection is therefore awakened (e.g. purchases in the early days of the virus caused a lack of supply of acetaminophen in pharmacies). At the same time, the uncertainty of the situation provokes a normative behaviour that ensures the guidelines established by specialists are followed (“We will stop it if you help and listen to our professionals”), and behaviour is directed to act for the common good and maintain physical distancing from others [5]. All these messages have been created to promote beneficial changes in behaviour among members of the global community; in this sense, personal, descriptive, and injunctive normative messages had been analysed on fliers as effective tools for promoting pandemic response behaviours in Italy doing the norms more salient [6]. But these mass media messages must be understood from the perspective of an ecological model of health, that is, a model in which the core characteristics of individuals must be considered in interaction with the special pandemic circumstances [7]. For example, Van Bavel et al. [8] highlighted the interaction between individual, social, and cultural contexts that could influence engagement in some changes of behaviour. Hence, individuals adapt their social values to special conditions, like the demands of social isolation, where security and compliance values are given greater importance in the face of any event that could be threatening–more so than any other values, such as power or self-direction.

Certain social psychology theories have proven to be effective in explaining different social behaviors and can help us explain the behaviour of citizens in the face of a declared state of emergency, such as the Covid-19 pandemic [8]. In sum, the present investigation aims to evaluate the importance of beliefs in self-efficacy and confidence in collective actions, in addition to the level of information, as well as social values, in relation to the individual behaviours carried out for self-protection and the individual behaviours performed to follow the physical distancing norms throughout the first 10 days after the alarm period decreed by the Spanish government.

### Risk information seeking, self- and collective efficacy and social values

Due to globalized connectivity, the world has verified how the virus can spread at great speed to the entire globalized world, making any citizen from any remote place feel vulnerable to the threat, even though the infectious disease was initially somewhat localized in China. Half of the global population lives in cities, which favours the spread of viruses; indeed, global cities seem to make us more vulnerable to the threat. For this reason, following the practical guidance produced by the World Health Organization [1], an initial measure that countries have adopted has focused on normative behaviours based on the confinement of cities, social isolation, and the closing of borders (“physical distancing methods of isolation and quarantine”). The second measure has focused on self-protection behaviours for citizens, the use of gloves, masks, and hand washing, in order to “break the chain of transmission and contain the outbreak” [9]. To be precise, due to the perception of scarcity of these protective items, it is possible that individual behaviour that was initially focused on self-care behaviour became a consumption behaviour guided by selfishness or irrational fears of shortage [10]. Through social dilemma scenarios, Kollock [11] analysed individual behaviour when collective resources were perceived as scarce: in the face of scarcity, individuals tend to use competitive, selfish strategies that secure their own resources.

Under uncertainty scenarios, *risk information seeking* is an important variable for coping with vulnerability to disease. Thus, knowledge about the disease’s spread, the number of people affected, and the number of deaths in each area is important in regard to perceived risk–both in terms of the velocity and amplitude of the disease and the probability of being infected and suffering with the disease. The Planned Risk Information Seeking Model (PRISM [12]) has been applied under other health risk scenarios with higher levels of uncertainty, such as the Ebola outbreak [13] and the outbreak of the Zika virus [14]. Risk perception was associated with the search for related subjective norms, as well as with the perception of a lack of information. This perception causes individuals to stay better informed on a daily basis [14]. Therefore, we expect individuals characterized by risk information seeking behaviours to develop more behaviours for coping with Covid-19.

On the one hand, social cognitive theory focuses on the confidence that both individuals and collectives have that they will be able to carry out a specific behaviour in a specific situation [15]. Over the years, self-efficacy beliefs have been shown to play an important role as predictors of behaviours in different domains (e.g. health, academic, occupational) [15]. Once the focus is applied to pandemic situations and the demand directed to citizens to develop different self-care behaviours, the conceptualization of self-care self-efficacy beliefs must be revised. As a short summary, Mak and colleagues [16] defined self-care self-efficacy as the confidence a person has in their ability to perform relevant self-care activities, while Eller and colleagues [17] added to this definition dual competencies: perceived confidence in carrying out self-care behaviours as well as carrying out the self-care activities demanded by the community. Hence, while Lorig and Holman [18] point out that self-management behaviours are related to the degree to which patients have the confidence to follow the norms advised by their physician, in a pandemic situation, self-management is related to the extent to which individuals have the confidence to follow the norms decreed by political regulations on social isolation. Therefore, we believe it is necessary to evaluate both the self-care self-efficacy judgment that individuals have to follow the social isolation norms set by the government in the face of the state of alarm (*social isolation management self-efficacy*) and the confidence they have in their ability to develop self-protective behaviour that allows them to protect themselves and their family (*self-protection self-efficacy*).

Likewise, collective behaviour in which trust in others is extolled, as well as belief in the *collective efficacy* in following the rules and stopping the virus, must be considered. In this regard, Sampson [19] supported the relationship between collective efficacy and community safety, where neighbourhood collective efficacy is based on the trust in shared expectations for specific actions. Fong and Chang [20] highlighted the relevance of collective efficacy of a community during the outbreak of a pandemic, such as the severe acute respiratory syndrome (SARS) outbreak in 2003. They defined collective efficacy based on Hardin’s [21] conceptualization of trust as “rational expectations of the behaviour of the trusted”. Fong and Chang [20] found that community actions were positively related to the level of collective efficacy measured by trust in shared actions aimed at coping with the virus. Another hypothesis is that those individuals with higher perceptions of both self-efficacy judgments (social isolation management and self-protection self-efficacy) and collective efficacy will develop more appropriate behaviours for coping with Covid-19, in terms of both self-care and normative behaviours associated with social isolation.

On the other hand, in a pandemic situation, *personal and social values* can be adapted to these extraordinary life circumstances [22]. Basic personal values are cognitive representations that define the way people perceive what is important in their life [23]. According to the Schwartz [23] model, the values are structured circularly along different dimensions corresponding to values that are universally grouped [24]. For example, we would find a dimension that groups individual interests (self-direction, stimulation, hedonism, and power), as opposed to a dimension that groups collective interests (conformity, tradition, benevolence, and universalism). Therefore, the circular structure of personal and social values proposed by Schwartz [23] suggested that self-transcendence and conservation are dimensions oriented toward a social focus, while openness to change and self-enhancement are oriented toward a personal focus. Furthermore, conservation and self-enhancement values are oriented toward self-protection, while self-transcendence and openness to change are oriented toward growth. Additionally, Schwartz [23] affirmed that people with high values of self-enhancement show more concern about threats to the self, while those with high values of self-transcendence show greater concern about threats to the community or society more broadly [24]; therefore, people can find a balance between values that allow them both greater individual and collective well-being. In the same vein, Callaghan [25] stated that in pandemic scenarios, the tension between “selfishness and altruism” is equivalent to the values in terms of tension between “self-transcendence and self-enhancement”.

Not many studies have analysed the relationship between social values and citizen behaviour in the context of a global pandemic. Daffin [26] analysed the relationship between social values and individual and collective behaviours in a simulation of a global pandemic that produced a public health crisis. Even though values are relatively stable and tend to serve as consistent guiding systems for behaviour, depending on the specific context and demands of the situation, the function of values can be modified, just like needs and motivations [22]. Daffin [26] concluded that in one sense, the pandemic activates *micro-level values* where worries are directed toward everything that affects one’s own safety and that of one’s immediate family, friends, and neighbourhood–that is, conservation values. In another sense, the pandemic activates *macro-level values*, safety, and health as life domains, the self-transcendence values. In the present study, another hypothesis is formulated on the basis that individuals who hold high self-transcendence values (e.g. universalism-societal concern) will develop fewer behaviours oriented to appeasing their self-interested needs or selfishness, while those individuals who hold values oriented toward conservation (e.g. personal and national security and conformity) will direct their actions toward following governmental norms of isolation and confinement related to physical distancing behaviours.

### The current study

In sum, the present study proposes as its main objective the analysis of psychosocial variables related to the performance of both physical distancing behaviours and self-interested consumption behaviours carried out during the first 10 days of confinement in Spain, which were characterized by an increasing number of deaths and uncertainty about the future development of the pandemic in proximal and distal contexts. To achieve this objective, socio-demographic and motivational variables related to the search for information on risks, the confidence in self- and collective capacity to cope with the pandemic, and the main social values that guide behaviour (conservation, self-transcendence, openness, and self-improvement) were analysed. Given the novelty of the behaviour analysed, the first specific objective (O1) focused on developing specific evaluation scales for both self-efficacy and collective efficacy, as well as coping behaviours in the face of the pandemic. The second specific objective (O2) focused on evaluating the relationship between all the variables analysed (socio-demographic, psychological, and behavioural), as well as the change in behaviours as the disease spread. The third specific objective (O3) focused on developing an explanatory model of the behaviours studied in facing Covid-19. For the third objective, it is proposed that the direction of the relations be in accordance with the scientific background and the literature reviewed, and with the cognitive-affective personality system (CAPS). The CAPS [27] is a widely accepted and empirically validated model oriented toward the behavioural explanation that reveals how dispositional variables (such as social values) influence self-regulatory variables (such as self- and collective efficacy), which in turn affect behaviour.

## Method

### Participants and procedure

A total of 1,324 people, with a mean age of 28.92 years and a standard deviation of 12.68 (range 18 to 83 years), participated in this research. Of these participants, 74.4% were women (n = 985) and 25.6% men (n = 339). Participants reported on the number of people with whom they shared a home. In total, they shared a home with an average of 2.50 people (SD = 1.25), specifically: 5.1% did not share with anyone; 17.1% shared with one person; 26.3% shared with two people; 32.6% shared with three people; 14.3% shared with four people; 2.9% shared with five people; and 1.7% shared with six or more people. Participants were residents across almost the entire Spanish territory: 23.3% Córdoba; 15.5% Salamanca; 11% Canary Islands; 7.9% Almería; 5% Madrid; 6.9% other places in Castilla and León; 3.5% Valencia; 2.9% Barcelona; 2.9% Seville; 2.8% Malaga; 2.3% Extremadura; 1.6% Galicia; and 14.4% other places. The number of citizens in the areas in which they were confined varied from two to six million citizens.

The data were collected online from different psychology teachers who distributed the link among the students, which in turn was shared on different social networks. Specifically, the text accompanying the survey said:

From different universities we ask for your collaboration by answering a short survey to find out how Spanish society is facing the unprecedented situation generated by the expansion of Covid-19. It won’t take more than 10 minutes of your time. Keep in mind that the survey is anonymous, that the data will be treated as a whole and not individually, and that there are no correct or incorrect answers. We only want to know your opinion. So it is important that you answer honestly. Your participation is voluntary, and you can withdraw from the study whenever you want.

After giving their informed consent, the questions began. They were initially focused on socio-demographic factors and, subsequently, on a series of items that allowed them to respond to the objectives of the study. The study was not reviewed nor approved by any institutional review board (ethics committee) before the study began because the Spanish Ministry of Science and Innovation requires this kind of revision and approval only when the studies imply clinical human or animal experimentation.

The message was shared on Monday, March 16, the day on which social isolation measures began. On that day, Spain registered a mortality of 21 deaths; 10 days later, on Wednesday March 25, 728 deaths were recorded. For the first 10 days of isolation, data were collected every day, and a total of 3,136 deaths were recorded (number of deaths per day: 21, 188, 101, 169, 215, 344, 394, 462, 514, 728). The peak of the spread of the virus had almost been reached.

### Measures

#### Socio-demographic variables

The socio-demographic variables included age, sex, number of people who shared their home, number of citizens in the town in which they lived in the present confinement state (the mean was 495,597.35 citizens), whether they knew at least one person affected with coronavirus (13.1%, n = 173 citizens knew personally a person affected by Covid-19), and whether they had a family member affected by coronavirus (4.5%, n = 60 citizens had a family member affected by Covid-19).

#### Risk information seeking

Six questions were developed to assess the extent to which people knew the information on coronavirus cases in their cities and communities (e.g. “Today, do you know how many positive cases of coronavirus there are in the city where you reside during the emergency situation?”). The questions were related to the number of positive cases, deaths caused by the virus, and recoveries from coronavirus in their cities and communities. If the answers were positive, the participants had to indicate the relevant figure. A summation measure was created with the total number of positive responses (ranging from 0 to 6).

#### Self- and collective efficacy in coping with the Covid-19 pandemic

This measurement of self- and collective efficacy was created following the instructions of the guide to constructing self-efficacy scales [28]. Following this guide, the specific recommendations produced by the Spanish government were analysed (https://www.mscbs.gob.es/profesionales/saludPublica/ccayes/alertasActual/nCov-China/ciudadania.htm). The individual perception of the capacity to carry out each of the specific self-care and normative acts was evaluated using 13 items (e.g. “To what extent do you feel capable of remaining at home for the period determined by the government?”). Participants were required to reflect on their levels of confidence using a six-point scale, where response scores ranged from 1 = not at all confident to 6 = totally confident. The Cronbach’s alpha for this scale was .84.

After these questions, 12 items were presented to evaluate the collective perception of the capacity to carry out each of the specific self-care and normative acts (e.g. “To what extent do you have confidence in the citizens remaining isolated from any social contact with friends, family, neighbours …”). Participants were required to reflect on their levels of confidence using a six-point scale, where response scores ranged from 1 = not at all confident to 6 = totally confident. The Cronbach’s alpha for this scale was .91.

#### Basic personal values

A short version of the Portrait Values Questionnaire, developed by Schwartz [29], was utilized to evaluate the cognitive representation of desirable, trans-situational goals [30], so 28 portrait values were presented to evaluate individual desires that point implicitly to the importance of a value (e.g. “He/she believes that people should do everything they are told to do. He/she believes that people should always follow the rules, even when nobody is watching them”). A validated Spanish version of the questionnaire was used [31]. For each portrait, respondents indicated how similar the person was to themselves on a scale ranging from 1 = not like me at all to 6 = very much like me. Respondents’ own values are inferred from the implicit values of the people they describe as similar to themselves. The factors evaluated were: *conservation values*, with 10 items and a reliability of 0.87 (personal security, national security, and conformity); *self-transcendence values*, with eight items and a reliability of 0.92 (benevolence, universalism societal concern, and universalism environmental concern); *openness values*, with five items and a reliability of 0.78 (self-direction actions and stimulation); and *self-enhancement values*, with five items and a reliability of 0.73 (hedonism and power). According to the Schwartz model [29], self-transcendence and conservation are dimensions oriented toward social focus, while openness to change and self-enhancement are oriented toward personal focus. Conservation and self-enhancement values are oriented toward self-protection and anxiety avoidance, while self-transcendence and openness to change are oriented toward becoming anxiety-free.

#### Behaviours for coping with Covid-19

Following the government’s recommendation, a list of eight behaviours related to the pandemic situation was prepared. Participants respond to the level at which they have performed the behaviours related to self-protection (e.g. “bought medications in case they could be needed in future”) and normative behaviours related to physical distancing or isolation (e.g. “avoided shaking hands with others”). Participants were required to reflect on their levels of confidence using a six-point scale, where response scores ranged from 1 = never to 6 = always. The Cronbach’s alpha for the whole scale of eight items was 0.76.

### Statistical analyses

First, the whole sample was split into two random subsamples. Participants in both subsamples had similar socio-demographic characteristics. The data obtained from random sample I (N = 643) were subjected to exploratory factor analysis (EFA) with varimax rotation to evaluate the underlying factor structure in each variable, and once the reliability was confirmed as acceptable, the dimensions were created from the mean (O1). This was tested in random sample II (N = 681) using confirmatory factor analysis (CFA). The suitability of using EFA was assessed using Bartlett’s Test of Sphericity (BTS) and the Kaiser–Meyer–Olkin (KMO) statistic. A KMO value of .50 or higher is considered acceptable for a satisfactory EFA to proceed [32]. For BTS, a p value ≤ .05 serves as the criterion for indicating that implementation of EFA is possible [33]. As for CFA, we evaluated model fit using the following statistics: chi-squared (χ^2^), the χ^2^/df ratio, the root mean square error of approximation (RMSEA), the Goodness-of-Fit Index (GFI), the Adjusted Goodness-of-Fit Index (AGFI), the Normed Fit Index (NFI), the Confirmatory Fit Index (CFI), IFI, and the Tucker-Lewis index (TLI). For model evaluation, we followed Schermelleh-Engel, Moosbrugger, and Müller’s recommendations [34]: acceptable model fit is indicated by χ2/df equal to or less than 3, RMSEA lower than .08, with a confidence interval (CI) close to RMSEA, GFI and NFI equal to or higher than .90, AGFI between .85 and .90, CFI and TLI equal to or higher than .95, and IFI. Good model fit is indicated by χ2/df equal to or less than 2, RMSEA between 0 and .05, with CI close to RMSEA, GFI and NFI equal to or higher than .95, AGFI higher than .90, CFI and TLI equal to or higher than .97, and IFI.

Then, correlation analyses were carried out to check the relationships among socio-demographic, psychological, and behavioural variables. Next, univariate analyses of variance (ANOVAs) and repeated-measures ANOVAs were performed to check the differences between sex and the level of risk seeking information with behaviours analysed over time (O2). Lastly, structural equation modelling (SEM) was created from theory-based assumptions where the directionality of the relationship between variables could be supported (O3). The same indices and cut-off recommendations were used as those for testing CFA.

## Results

### Exploratory and confirmatory factor analysis of new measures created (O1)

#### Self-efficacy in coping with Covid-19 pandemic

Before proceeding with the correlational analysis to test the relationship between all the study variables, an EFA was carried out, which identified two factors that explained 49.03% of the variance. The KMO index (.88) and BTS (χ^2^ = 2361.34; DF = 66; *p* < .001) supported the use of EFA. Table 1 shows the loading of each item in each factor and the factors’ reliability. The final bidimensional structure was checked by a CFA (see S1 Fig in S1 Appendix), and the bifactorial model showed a good model fit (χ^2^ (DF = 33) = 110.66, *p* = .000; GFI = .986; AGFI = .968; NFI = .975; CFI = .982; IFI = .982; TLI = .965; RMSEA = .042 (Lo 90 = .034; Hi 90 = .051).

**Table 1:** Items for self-efficacy beliefs in coping with Covid-19. An exploratory factor analysis shows the item loadings in each dimension, as well as mean and standard deviation.

Items (13 items, α= .84) General item: *1*. *How confident are you that your behaviour can help curb the virus*? *What level of confidence do you have that you will be able to successfully perform the following actions*?	Social Isolation Management Self-efficacy	Self-protection Self-efficacy	Mean (SD)
3. I feel able to keep myself isolated from any social contact (friends, family, neighbours …)	**.86**	.11	4.74 (1.37)
5. I feel able to maintain at least the minimum social contact with friends, family, neighbours …	**.84**	.16	5.02 (1.21)
2. I feel able to stay home at least for the period determined by the government	**.82**	.08	5.13 (1.15)
13. I feel able to organize my life (work, leisure …) at least for the duration of the confinement	**.58**	.33	5.06 (1.14)
4. I feel able to wash my hands with soap for at least 20 seconds as often as necessary	.19	**.61**	5.36 (0.96)
10. I feel able to follow all the rules and instructions of the professionals at all times (coughing at the elbow, not touching my face …)	.29	**.64**	4.96 (1.01)
12. I feel able to offer my help to the most needy or vulnerable people in this alarming situation	.07	**.61**	5.18 (1.06)
7. I feel able to keep at least a metre away when talking to others	.45	**.53**	5.11 (1.09)
8. I feel able to ask those around me to follow the standards set by the government	.40	**.51**	5.28 (1.01)
9. I feel unable to share on social networks all the information that I receive about Covid-19 without first verifying its authenticity	-.25	**.53**	5.42 (1.01)
6. I feel able to organize my meals with what I have at home to avoid going outside unnecessarily	.44	**.45**	5.07 (1.01)
11. If I must go out to go shopping, I feel able to comply with the rule of doing it alone (or in the company of someone who is in my charge and cannot stay alone at home)	.29	**.56**	5.57 (0.79)
% explained variance Reliability	37.22% α = .83	11.81% α = .74	

#### Collective efficacy in coping with the Covid-19 pandemic

The EFA showed one factor that explains 54.44% of the variance. The KMO index (.93) and BTS (χ^2^ = 3976.54; DF = 55; *p* < .001) supported the use of EFA. Table 2 shows the reliability and loading of each item. The final unidimensional structure was checked by a CFA (see S2 Fig in S1 Appendix), and the unifactorial model showed a good model fit (χ^2^ (DF = 33) = 150.98, p = .000; GFI = .982; AGFI = .956; NFI = .982; CFI = .986; IFI = .986; TLI = .971; RMSEA = .052 (Lo 90 = .044; Hi 90 = .061)

**Table 2:** Items for collective efficacy beliefs in coping with Covid-19. An exploratory factor analysis shows the item loadings, as well as mean and standard deviation.

Items (12 items, α = .91) General item: *1*. *How confident are you that the behaviour of others can help curb the virus*?	Load	Mean (SD)
10. I trust that people follow all the rules and instructions of the professionals at all times (coughing at the elbow, not touching their face …)	**.84**	3.65 (1.10)
5. I trust that people maintain the minimum social contact with friends, family, neighbours …	**.83**	3.59 (1.17)
3. I trust that people remain isolated from any social contact (friends, family, neighbours …)	**.80**	3.63 (1.15)
2. I trust that people are staying at home for the period determined by the government	**.77**	3.86 (1.13)
11. I trust that people who go out shopping follow the rule of going out alone	**.76**	3.89 (1.24)
4. I trust that people wash their hands with soap for at least 20 seconds as often as necessary	**.76**	3.62 (1.25)
7. I trust that people keep at least a metre away when talking to others	**.71**	3.75 (1.25)
6. I trust that people organize their meals with what they have at home to avoid going outside unnecessarily	**.71**	3.46 (1.26)
8. I trust that people ask those around them to follow the standards set by the government	**.74**	4.01 (1.16)
9. I trust that people are unable to share on social networks all the information that they receive about Covid-19 without first verifying its authenticity	**.57**	2.40 (1.30)
12. I trust that people offer their help to the most needy or vulnerable people in this alarming situation	**.55**	4.26 (1.19)

#### Behaviours for coping with the Covid-19 pandemic

Once the first item had been excluded from the analysis because of the low loading, the EFA showed two factors that explain 67.66% of the variance. The KMO index (.75) and BTS (χ^2^ = 1981.21; DF = 21; *p* < .001) supported the use of EFA. Table 3 shows the reliability and loading of each item in each factor. The proposed factorial structure was checked by a CFA (see S3 Fig in S1 Appendix), and the bidimensional model showed a good model fit (χ^2^ (DF = 5) = 14.18 *p* = .01; GFI = .997; AGFI = .983; NFI = .996; CFI = .998; IFI = .998; TLI = .990; RMSEA = .037 (Lo 90 = .015; Hi 90 = .061).

**Table 3:** Items related to developed behaviours in the context of the pandemic, means and standard deviation. Factorial structure from the exploratory factor analysis, item loading, percentage of explained variance, and reliability of each factor.

Items (8 items, α = .76; *M* = 2.99, Sd = 0.97) Item related to information seeking “1. I follow the information related to Covid-19 on TV, radio, newspapers, social networks …” (*M* = 4.86; Sd = 1.12)	Physical distancing behaviours *M* = 2.48 Sd = 1.17	Self-interested consumption behaviours *M* = 3.01 Sd = 1.55	Mean (SD)
7. Since the government declared the state of alarm, I have avoided shaking hands with others	**.94**	.08	3.23 (1.75)
8. Since the government declared the state of alarm, I have avoided kissing when greeting others	**.93**	.07	3.33 (1.80)
6. Since the government declared the state of alarm, I have kept more than a metre away from others	**.83**	.24	2.69 (1.56)
3. I have bought medications in case I need them (paracetamol, alcohol, …)	.13	**.81**	2.45 (1.58)
5. I have bought alcohol or disinfectant gel in case I needed them	.14	**.77**	2.38 (1.67)
4. I have bought masks in case I need them	-.01	**.80**	1.64 (1.29)
2. Since the isolation period was declared, I have made the necessary purchases to be able to stay at home without leaving the house for 15 days	.33	**.46**	3.07 (1.59)
% explained variance Reliability	44.22% α = .90	23.44% α = .72	

### Relationship between all the variables analysed and the change in behaviours as the disease spread (O2)

#### Relationship between self- and collective efficacy with behaviours in coping with Covid-19

Table 4 shows the positive and significant correlation between self- and collective efficacy with both behaviours identified for coping with the Covid-19 pandemic: self-interested consumption and physical distancing behaviours. Additionally, univariate analysis shows that the personal confidence to have the ability to develop successful self-protection behaviours is significantly higher than the ability to execute social isolation management (*F* (1,1323) = 132.70, *p* < .001, eta ^2^ = 0.09). On the other hand, univariate analysis showed higher confidence in personal self-efficacy beliefs, ahead of the confidence in collective beliefs in coping successfully with the Covid-19 pandemic (*F* (1,1323) = 3354.94, *p* < .001, eta ^2^ = 0.72).

**Table 4:** Means and standard deviations of general self-efficacy in coping with Covid-19 and the two factors of self-efficacy (self-protection and social isolation management) and collective efficacy.

Self- and collective efficacy	Coping behaviours	Self-interested consumption behaviours	Physical distancing behaviours	Information seeking	Mean (SD)
Self-efficacy in coping with Covid-19	.28**	.12**	.29**	.25**	5.16 (0.62)
Self-protection self-efficacy	.25**	.10**	.26**	.23**	5.25 (0.58)
Social isolation management self-efficacy	.25**	.11**	.25**	.21**	5.02 (0.89)
Collective efficacy	.11**	.08**	.08**	.12**	3.72 (0.85)

Correlations between self- and collective efficacy with behaviours related to coping with the Covid-19 pandemic (* *p* < .05; ** *p* < .01).

**Fig 1:**
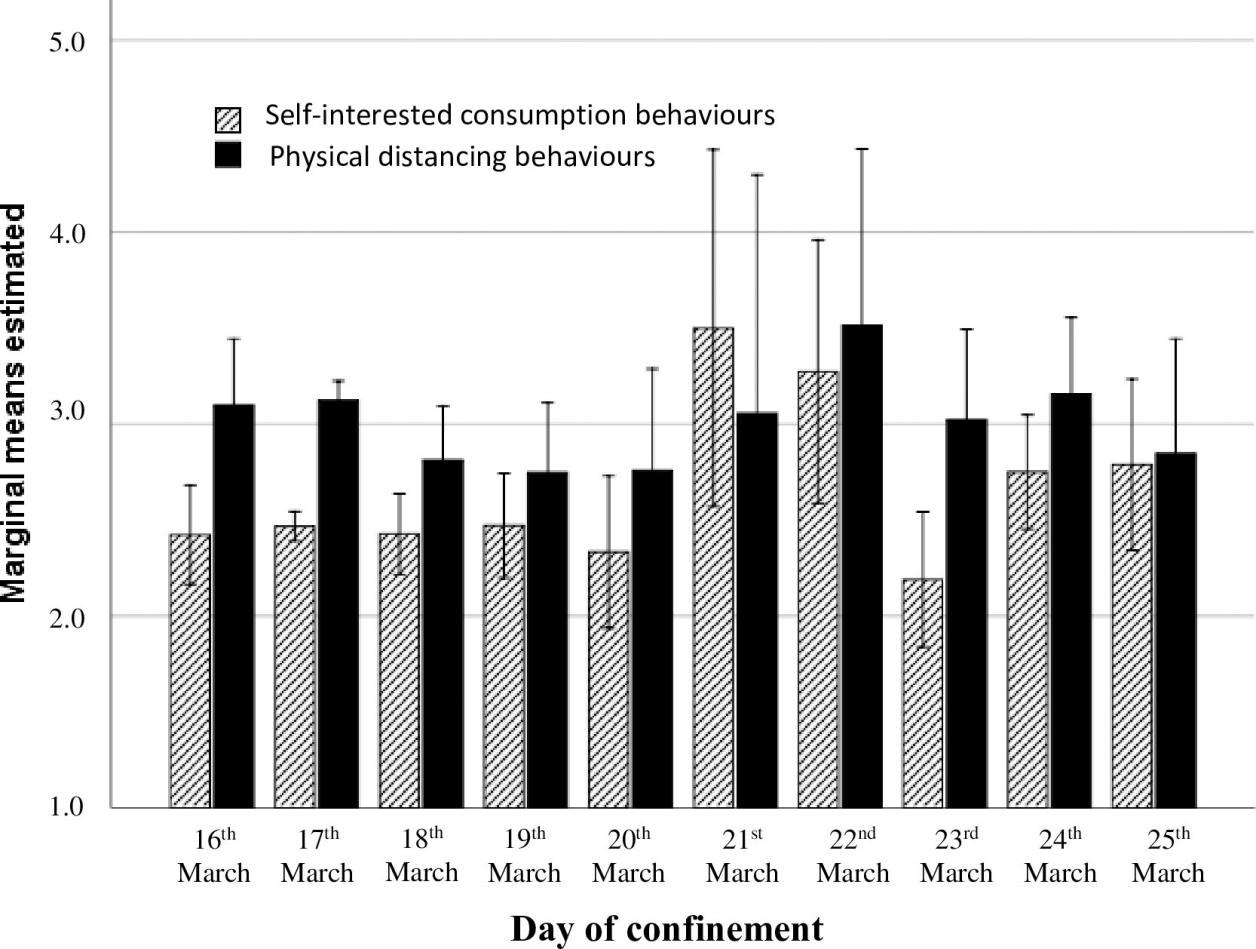
Means and standard distribution for both behaviours studied in coping with Covid-19 (self-interested consumption and physical distancing behaviours) during the 10 first days of confinement in Spain.

In relation to the *behaviours developed to cope with the Covid-19 pandemic*, a univariate analysis showed that individuals developed significantly more behaviours to maintain physical distancing from others (*F* (1,1323) = 165.54, *p* < .001, eta ^2^ = 0.11; *M* = 3.06, *SD* = 1.55) than behaviours related to self-interested consumption (*M* = 2.48, *SD* = 1.17). Furthermore, a repeated-measures analysis showed that this difference remained stable throughout the first 10 days (*F* (1,1314) = 11.32, *p* < .001, eta ^2^ = 0.09), while the interaction between time and both behaviours did not show any significant effect (*F* (1,1314) = 1.51, *p* = .14, eta ^2^ = 0.01). In Fig 1, the mean and standard distribution for each behaviour can be observed for each day of confinement. The pattern was similar on weekdays, while at weekends, the standard distribution was higher, and on day 6, Saturday, the pattern had changed to higher self-interested consumption than physical distancing behaviours.

**Fig 2:**
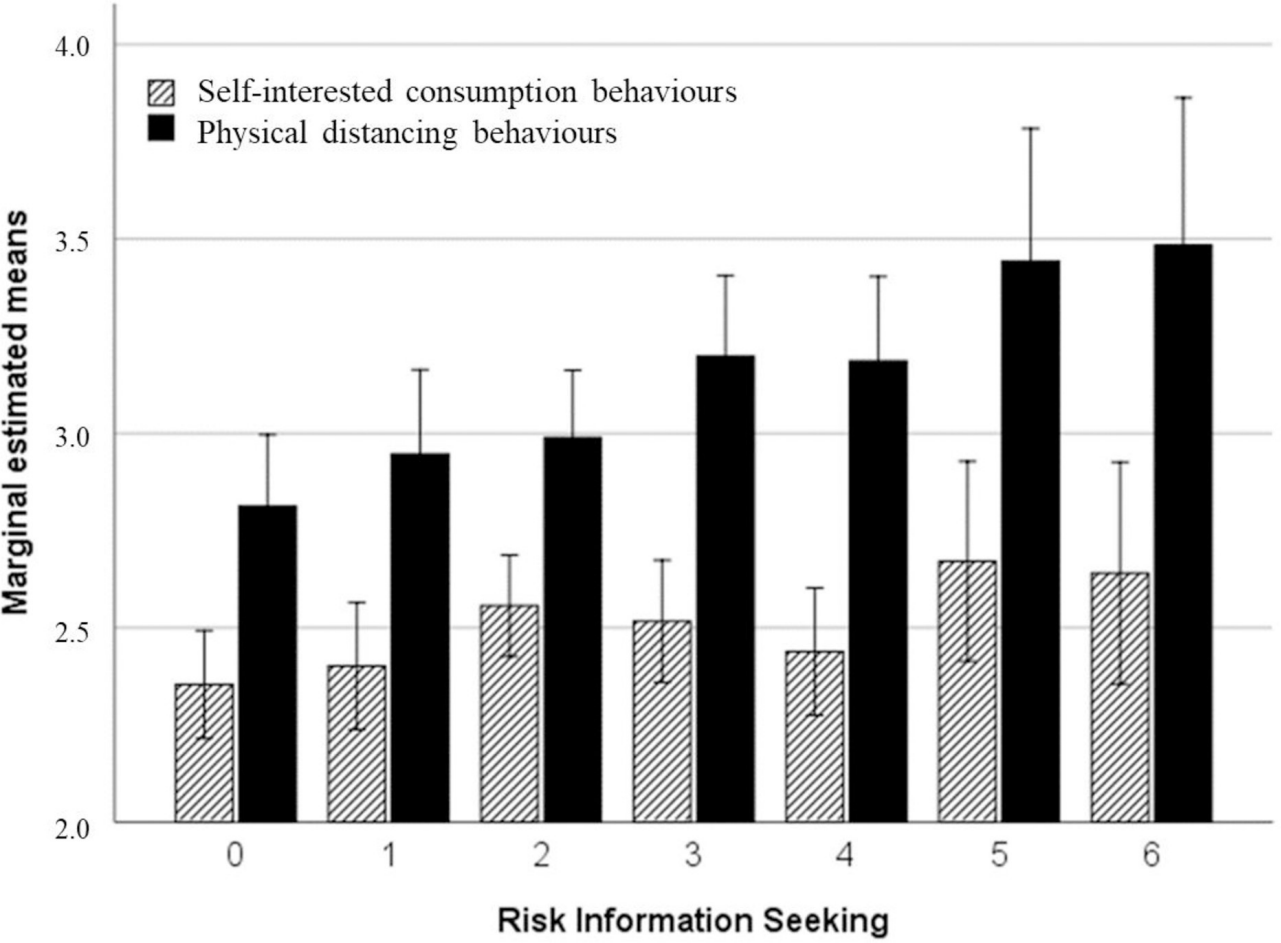
Means and standard distribution for both behaviours studied in coping with Covid-19 (self-interested consumption and physical distancing behaviours) along the risk information seeking distribution.

In relation to *risk seeking information*, univariate analysis showed that those participants with higher risk seeking information developed a high number of behaviours for coping with the Covid-19 pandemic (*F*(6,1317) = 3.31; *p* < .01; eta2 = .02) and those participants without any interest in risk seeking information showed significant differences in post hoc analysis from individuals who had some interest in risk information: DMS post hoc pair 0‒2 (*t* = -.19, *p* < .05), pair 0‒3 (*t* = -.26, *p* < .01), pair 0‒4 (*t* = -.21, *p* < .05), pair 0‒5 (*t* = -.45, *p* < .01), pair 0‒6 (*t* = -.45, *p* < .001). When the analysis was repeated, differentiating by the type of behaviour (see Fig 2), the univariate analysis showed that physical distancing behaviours increase as the pattern of searching for risk information increases (*F* (6,1317) = 3.56, *p* = .002, eta 2 = .02, pot = .95; DMS post hoc pair 0‒3 (*t* = -.39, *p* < .01), pair 0‒4 (*t* = -.38, *p* < .01), pair 0‒5 (*t* = -.63, *p* < .001), and pair 0‒6 (*t* = -.67, *p* < .001), while self-interested consumption behaviours did not experience any significant change as the search for information grew (*F* (6,1317) = 1.53, *p* = n.s.).

#### Relationship between personal and social values with behaviours for coping with Covid-19

Table 5 shows positive and significant correlations between conservation values and physical distancing behaviour and information seeking, while negative and significant correlations between self-transcendence values and self-interested consumption behaviours and information seeking were found. Additionally, a repeated-measures with post hoc analysis shows that self-transcendence values had significantly higher relevance (*F* (3,1321) = 827.35, *p* < .001, eta ^2^ = 0.65) than conservation (*t* = 0.70, *p* < .001), openness (*t* = 0.67, *p* < .001), and self-enhancement values (*t* = 1.04, *p* < .001); and self-enhancement values received lower relevance than other values (conservation: *t* = -0.34, *p* < .001, and openness (*t* = -0.38, *p* < .001).

**Table 5:** Means and standard deviations of conservation (personal security, national security, and conformity); self-transcendence (benevolence, universalism societal concern, and protecting environment); openness (self-direction action and stimulation); and self-enhancement values (hedonism and power).

Main social and personal values	Coping Behaviours	Self-interested consumption behaviours	Physical distancing behaviours	Information seeking	Mean (SD)
**Conservation**	.11**	**.05**	.09**	.18**	4.46 (0.87)
Personal Security	.12**	.05	.10**	.15**	4.61 (1.00)
National Security	.06*	.02	.06*	.16**	4.82 (1.09)
Conformity	.08**	.02	.07**	.17**	4.47 (0.98)
**Self-transcendence**	-.06*	-.10**	**-.01**	**.05**	5.15 (0.83)
Benevolence	-.02	-.04	-.01	.07*	5.14 (0.97)
Universalism Societal Concern	-.07**	-.11**	-.02	.03	5.24 (0.86)
Universalism Protecting Environment	-.04	-.09**	.02	.03	5.00 (1.04)
**Openness**	**-.05**	**-.03**	**-.05**	**-.01**	4.49 (0.92)
Self-Direction Action	-.07**	-.11**	-.02	.005	4.99 (0.96)
Stimulation	-.02	.02	-.05	-.02	4.16 (1.11)
**Self-enhancement**	**.02**	**.03**	**-.01**	**.05**	4.12 (0.89)
Hedonism	-.02	.02	-.04	.04	4.70 (1.03)
Power	.07*	.05	.06*	.03	3.23 (1.25)

Correlations between personal values and behaviours related to coping with the Covid-19 pandemic (* *p* < .05; ** *p* < .01).

Finally, in *relation to the socio-demographic variables evaluated*, the differences between men and women in all the psychosocial and behavioural variables were evaluated. The results showed that there were no significant differences in any of the behaviours evaluated, either in the collective efficacy or in the values of openness and self-enhancement; however, women showed significantly greater self-efficacy for both self-protection [*F* (1,1322) = 15.99, *p* < .001; *M*female = 5.29, SD = 0.58; *M*male = 5.14, SD = 0.67] and social isolation [*F* (1,1322) = 3.69, *p* = .05; *M*female = 5.05, SD = 0.86; *M*male = 4.94, SD = 0.96]. Likewise, women showed significantly higher values in conservation [*F* (1,1322) = 7.80, *p* < .01; *M*female = 4.50, SD = 0.85; *M*male = 4.34, SD = 0.91] and self-transcendence [*F* (1,1322) = 15.61, *p* < .001; *M*female = 5.21, SD = 0.81; *M*male = 5.01, SD = 0.86].

Age showed a significant and positive correlation with self-efficacy measures (self-protection: *r* = .09; *p* < .001; and social isolation management: *r* = .28; *p* < .001), collective efficacy (*r* = .14; *p* < .001), and conservation values (*r* = .07; *p* < .01). However, the correlation was significant and negative with openness (*r* = -.22; *p* < .001) and self-enhancement values (*r* = -.20; *p* < .001). In relation to the number of individuals sharing a dwelling, higher numbers of people sharing a dwelling correlated positively and significantly with self-interested consumption behaviour (*r* = .09; *p* < .001) and negatively with self-efficacy for physical distancing (*r* = -.06; *p* < .05). The number of inhabitants of the town or city where participants were living did not show any relationship with the other psychological variables analysed.

### Explanatory model of the behaviours studied in facing Covid-19 (O3)

**Fig 3:**
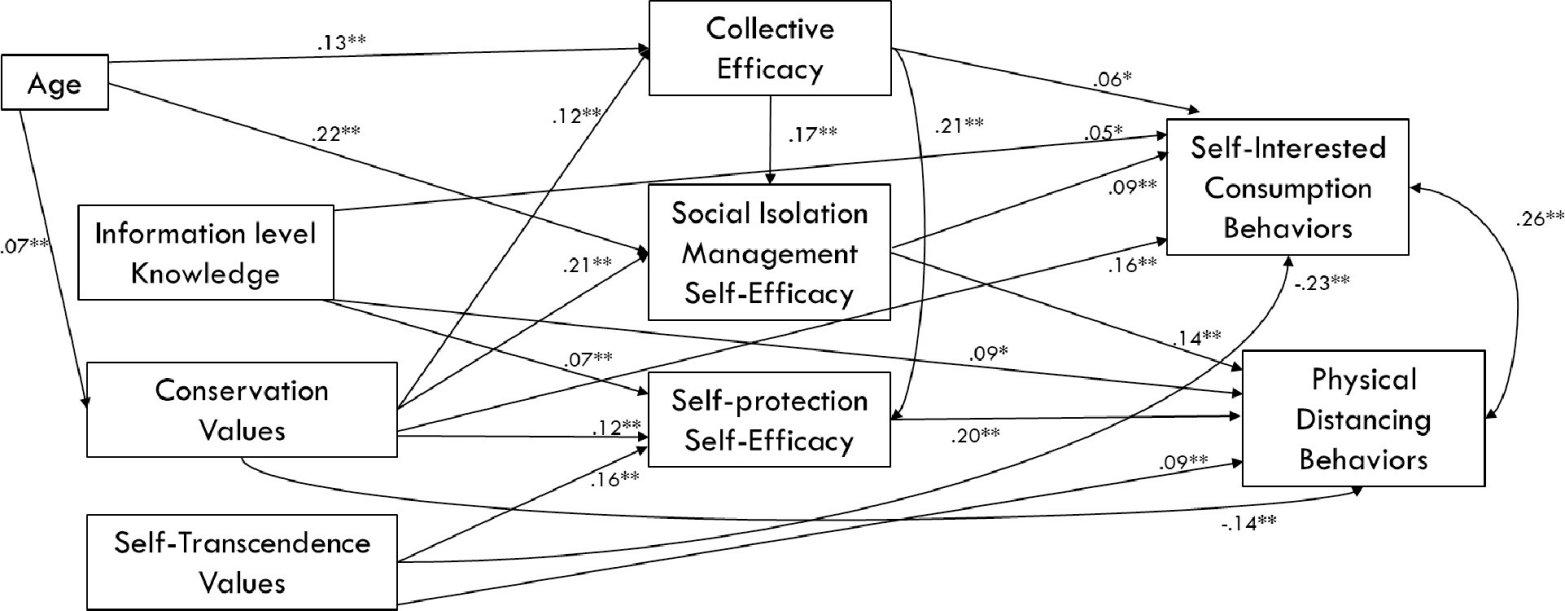
Structural equation modelling to explain the psychosocial variables involved in the development of behaviours studied (self-interested consumption and physical distancing) in coping with the Covid-19 pandemic in the first 10 days of confinement in Spain.

After analysing the relationships among all variables studied, and based on the research cited previously and the CAPS model [27], we developed a model of structural equations that allowed us to explain the variables that were related to both self-interested consumption behaviour and normative behaviour in maintaining greater physical distance from others. As Fig 3 shows, conservation and self-transcendence values were positively associated with self- and collective efficacy beliefs; and, in turn, these beliefs were positively related to both types of behaviour. However, as correlation analyses have shown previously, conservation values were positively related to both behaviours used to cope with the Covid-19 pandemic in the first days of confinement, while self-transcendence values were negatively related to them. The model showed adequate adjustments, and all the variables followed the expected direction [χ^2^ = 25.467; DF = 11; *p* = .008; GFI = .995; AGFI = .980; IFI = .992; TLI = .973; CFI = .992; RMSEA = .034 (Lo 90 = .017; Hi 90 = .051)].

## Discussion and conclusions

This research has tried to analyse the relationship among personal social values, self-efficacy for self-protection, and the management of social isolation and beliefs in collective efficacy with the development of certain specific behaviours. The observed behaviours included in the present research, when analysed in the contexts of previous pandemics, were directed both toward the protection of one’s own health through physical distancing [9] and at preventing self-interested behaviours directed at the exaggerated consumption of unnecessary products driven by an irrational fear or the perception of scarcity and the competitive behaviour of others [10]. A government-mandated shutdown is essential to stop the spread of the virus from day one. For this reason, knowing the variables that influence the maintenance of normative behaviours during the first 10 days of confinement, it may be essential to highlight motivational messages directed at the population to achieve greater regulatory adjustment and stop the spread of the virus.

Our results have shown that maintaining beliefs both in individual capacity and in the ability of the community to carry out actions that protect us from the virus exerts a direct positive influence on the development of protective behaviours. Furthermore, the results have shown that citizens have greater confidence in their own abilities to develop behaviours that help curb the virus than the confidence they place in others. This is in line with the research developed by Roser and Nagdy [35], which showed that we are much more optimistic about our own capabilities than about the capabilities of others. So when comparing the item assessing how much you think your behaviour or that of others can help stop the virus, we found significant differences (t (1323) = 10.25, p < .001), showing greater individual optimism (M = 5.05, SD = 0.98) than social optimism (M = 4.66; SD = 1.26). Therefore, as in the research carried out by Rosser and Nagdy [35], it seems that there is a certain mistrust in others that could lead to social pessimism. From the applied point of view, this result is also very important, since our data have shown that confidence in the ability of others or collective efficacy to stop the virus both directly and indirectly influences the individual behaviours of citizens. Therefore, health messages should also be aimed at building trust in others and thus avoiding social pessimism. At the cross-cultural level, it would also be interesting to analyse the differences between self- and collective efficacy found by Klassen [36] when comparing Asian and Western samples, so as to analyse the efficacy of messages aimed at self- or collective efficacy. The present study has not allowed for cross-cultural comparisons to be made regarding differences between self- and collective efficacy. It has, however, allowed us to analyse the relationship with the personal and social values analysed. Belief in one’s ability to stop the virus correlates with conservation values and not with openness values (*r* = .11, *p* < .001; *r* = .03, *p* = n.s., respectively), while the belief that the behaviour of others can help stop the virus correlates with the values of openness but not with those of conservation (*r* = .06, *p* < .05; *r* = .01, *p* = n.s., respectively). The relationship between collective efficacy and openness values remains positive and significant (*r* = .08, *p* < .01).

Likewise, conservation values based on maintaining one’s own security and national security as well as compliance positively influence people to carry out the regulatory behaviours decreed by the government based on maintaining physical distance from others. However, the values of self-transcendence, especially those related to universalism social concern, are those that prevent people from engaging in selfish purchasing behaviours that can trigger shortages or even panic or alarming situations that generate a greater crisis. The relevance of these results is highlighted by implications proposed by Callaghan [25], who affirmed that values are transmitted into, across and within individuals and groups and that the value system can give an alternative in managing pandemic situations.

As shown in the structural equation modelling, beliefs of self-efficacy and collective efficacy can act as mediating variables between conservation values and the normative behaviours followed. Therefore, since ability beliefs are more easily induced than values through motivating government messages, we believe they would be essential in the early days of confinement. Future research may explore the influence of persuasive messages directed at both the self and the community (e.g. “you” or “we can stay home and help stop the virus”), or persuasive messages directed at endorsing social comparison as a source of self-efficacy (e.g. “most of your neighbours stay at home”), which would help increase trust in others and avoid social pessimism. Previous research has shown that in situations of high uncertainty, such as a new pandemic, optimism is not related to monitoring healthy behaviours [37], while risk perception does directly affect behaviour. For this reason, we believe that generating confidence judgments in the personal and collective capacity to stop the virus is a central strategy in the early days of a pandemic.

In the same vein, the variable of risk information seeking has been shown to play an important role in explaining behaviour, because as citizens become more informed, they develop a greater number of normative physical distancing behaviours. These results found are in line with previous studies that have analysed the importance of searching for risk information regarding the Ebola virus [13] and the Zika virus [14]. In both studies, risk perception was associated with the monitoring of subjective norms.

As in the work of Morrison and Yardley [9], socio-demographic variables did not play an important role in predicting behaviours for coping with Covid-19. Males and females did not show significant differences in either of the behaviours studied. However, when we considered individual items (see Table 3), men were more likely than women to have bought masks (*F*(1,1322) = 6.19, *p* < .01; *M*male = 1.83, SD = 1.5; *M*female = 1.62, SD = 1.29) and less likely to avoid shaking hands with others (*F*(1,1322) = 5.94, *p* < .01; *M*male = 3.01, Sd = 1.76; *M*female = 3.28; SD = 1.74). Morrison and Yardley [9] also found more physical distancing behaviours in females than males. In terms of age, the results did not show any significant correlation with either of the studied behaviours. Again, when individual items were considered, age showed a positive and significant correlation with the declaration to follow the regulations on Covid-19 (*r* = .10, *p* < .001), maintain more than a 1 metre distance from others (*r* = .06, *p* < .05), and buy enough food to avoid leaving home (*r* = .10, *p* < .001); but it showed a negative and significant correlation with the declaration to have bought alcohol or disinfectant gel (*r* = -.11, *p* < .001). This was similar to previous results [9], where older adults were more likely to engage in certain types of preventive behaviours than others. Since older people are a risk group, a significant proportion of intervention programmes and communication messages should specifically target them, so that they develop physical distancing and protective behaviours to a greater extent than others.

### Future research and limitations

The first limitation of this study is related to the sampling strategy—essentially a snowball convenience sample—, it may be a potential source of bias since the sample was likely skewed towards users of social media, and because of the cross-sectional nature of the data collection, which does not ensure the directionality of the observed relations. Thus, the results of the explanatory model must be considered with precaution. However, there is sufficient underlying growth theory that supports the direction of the relation that we have proposed. The CAPS model [27], which explains that the personal variables influence the self-regulatory variables, which in turn affect behavior, is widely accepted and empirically validated. In any case, future longitudinal studies validating the results revealed in this research could be interesting. Another limitation is that our research could have evaluated other variables that could influence the two types of behaviour studied in the initial days of confinement–for example, the perception of social control exercised by neighbours over compliance with regulations in the community in which they reside [38]. Therefore, it would have been desirable to have a greater number of socio-demographic variables related to socio-economic status and educational level, both personal and in the area of residence. In our research, we do not know whether these variables could have influenced the monitoring of distancing behaviours or self-interested consumption, although we could anticipate that there would be some such relationship, since, for example, Reeves and Rothwell [39] point out that access to health resources for people with fewer socio-economic resources is worse than that of the economically privileged. Reeves and Rothwell [39] point out that there may be a greater effect of the virus in the most disadvantaged groups, as they cannot have the same access to the information and material necessary to follow personal hygiene and protection measures, such as masks, gloves, and sanitizing gels. Even overcrowding, which would mean living in a small house and not being able to maintain greater social distance, could influence having both a higher perceived risk and feeling more vulnerable to the economic consequences of the pandemic [40].

Other theoretical models could also help in understanding the motivation for normative behaviour in the early days of confinement. For example, according to the self-determination theory [41], it would be especially interesting to analyse the change that occurs over time in the motivation for compliance with standards ‒ going from the avoidance of penalization to the search for social recognition, the internalization of the norm, and the reaching of intrinsic motivation. Internal and intrinsic motivation would be the most permanent motivation for long-term commitment to behaviour in the face of the pandemic, and it would also be the motivation that would generate the greatest satisfaction in individuals. And of course, we could also consider the analysis of the emotional states that the pandemic produces in individuals (uncertainty, anxiety, fear, irritability, vulnerability, etc.), both in terms of behaviour in the face of the pandemic and in terms of the mental and physical health of citizens. In sum, Covid-19 is generating a significant number of investigations to try to explain and predict human behaviour–research that is necessary to prevent the spread of future pandemics and assess their effects on health and the associated socio-economic consequences.

In conclusion, since the growth of a pandemic is exponential, adherence to norms of physical distancing behaviour is essential in the early days, when very few cases of people infected with a virus are recorded. In this early stage, the level of uncertainty is high, and self-interested behaviours can also be registered in the event of a possible food shortage. Our research has shown that people maintain greater confidence in themselves and less confidence in others, although both judgments of self- and collective efficacy are essential for the development of certain specific behaviours during the first 10 days of confinement. Likewise, maintaining high conservative values directs citizens to adhere to normative self-protective behaviour, while self-transcendence values prevent selfish behaviours that can generate alarm among the population. Ultimately, those people who have shown a greater concern about the risk posed by the virus are those who have developed a greater number of normative self-protection behaviours, and they have not engaged in more selfish behaviour. Analysing the weight of motivational variables related to pandemic behaviour will help us manage the risk of future viruses.

## Supporting information

S1 Appendix (DOCX)

S1 File (SAV)
