# Supplementary material for: Social values, self- and collective efficacy explaining behaviours in coping with Covid-19: Self-interested consumption and physical distancing in the first 10 days of confinement in Spain
Source: PLoS One. 2020 Sep 17;15(9):e0238682. doi: 10.1371/journal.pone.0238682 (PMC7498046; doi:10.1371/journal.pone.0238682)

APPENDIX

**Figure S1.** Bi-dimensional structure for self-efficacy beliefs to cope with COVID-19 on two dimensions from a confirmatory factorial analysis: social isolation management self-efficacy and self-protection self-efficacy.

**Figure S2.** Uni-dimensional structure for collective efficacy beliefs to cope with COVID-19 in one uni-factorial dimension.

**Figure S3.** Bi-dimensional structure for behaviors in the context of the pandemic COVID-19 from a confirmatory factorial analysis: physical distancing behavior and self-interested consumption behaviours

Figure S1.


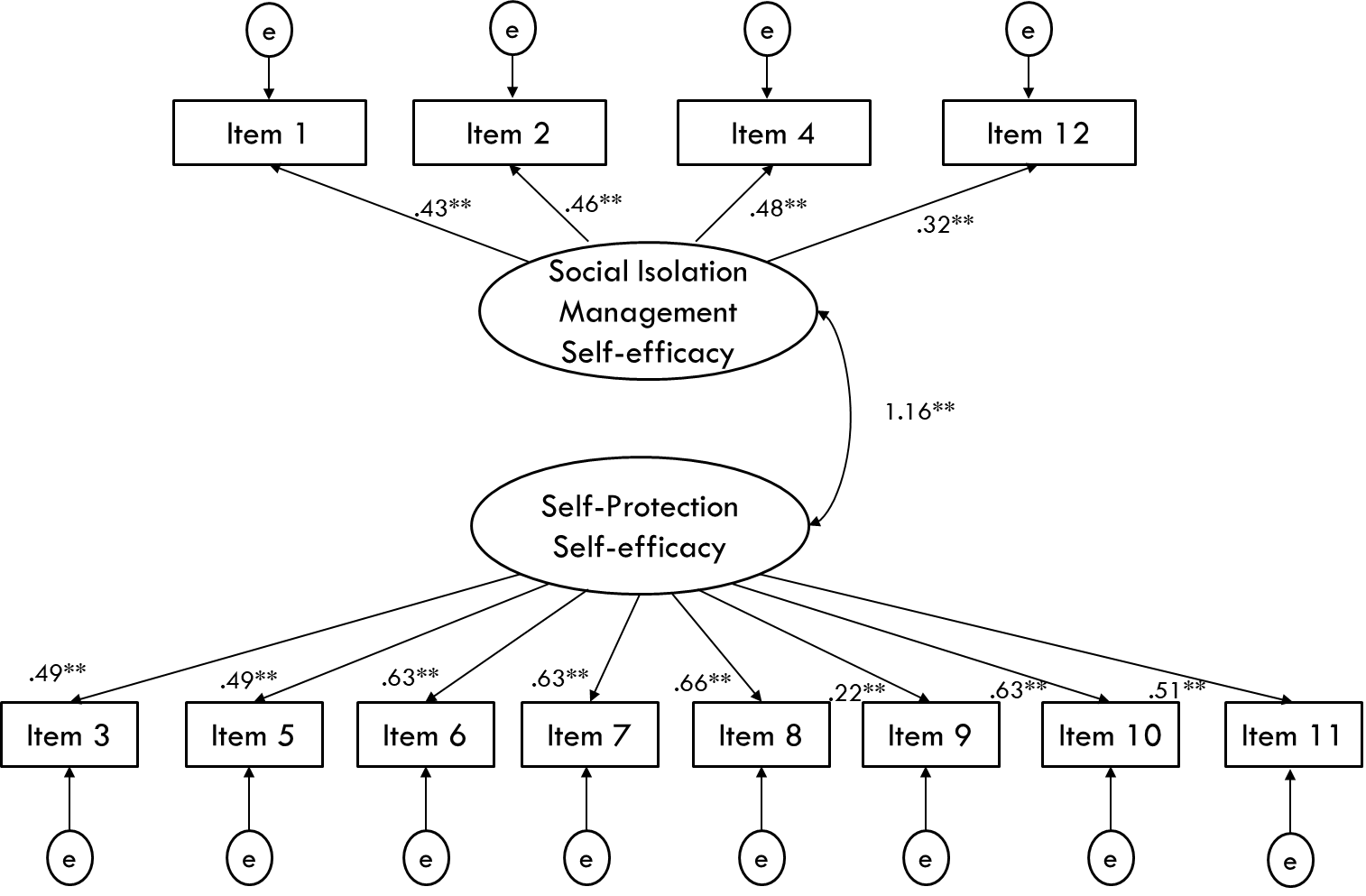


Figure S2.


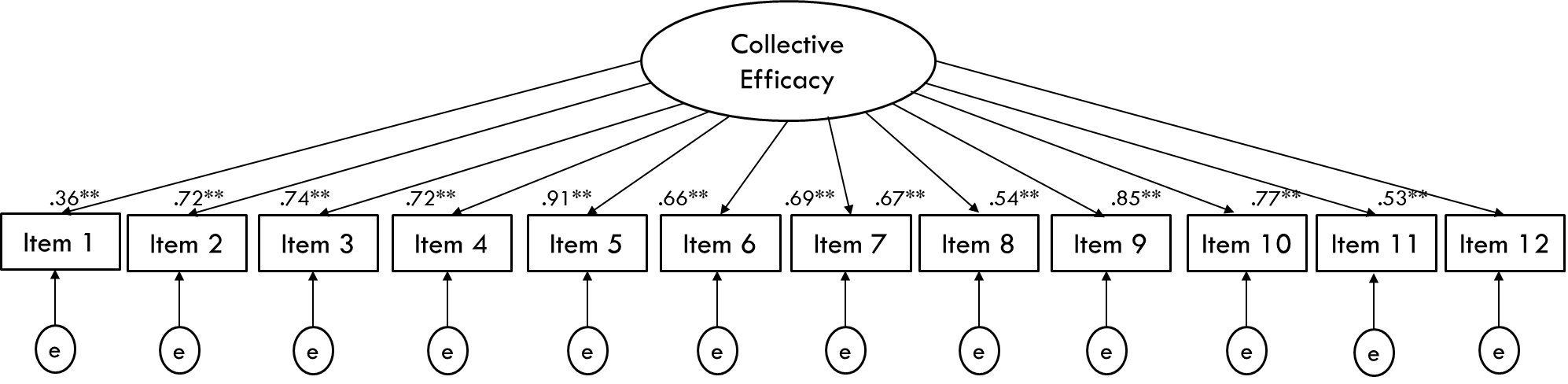


Figure S3.


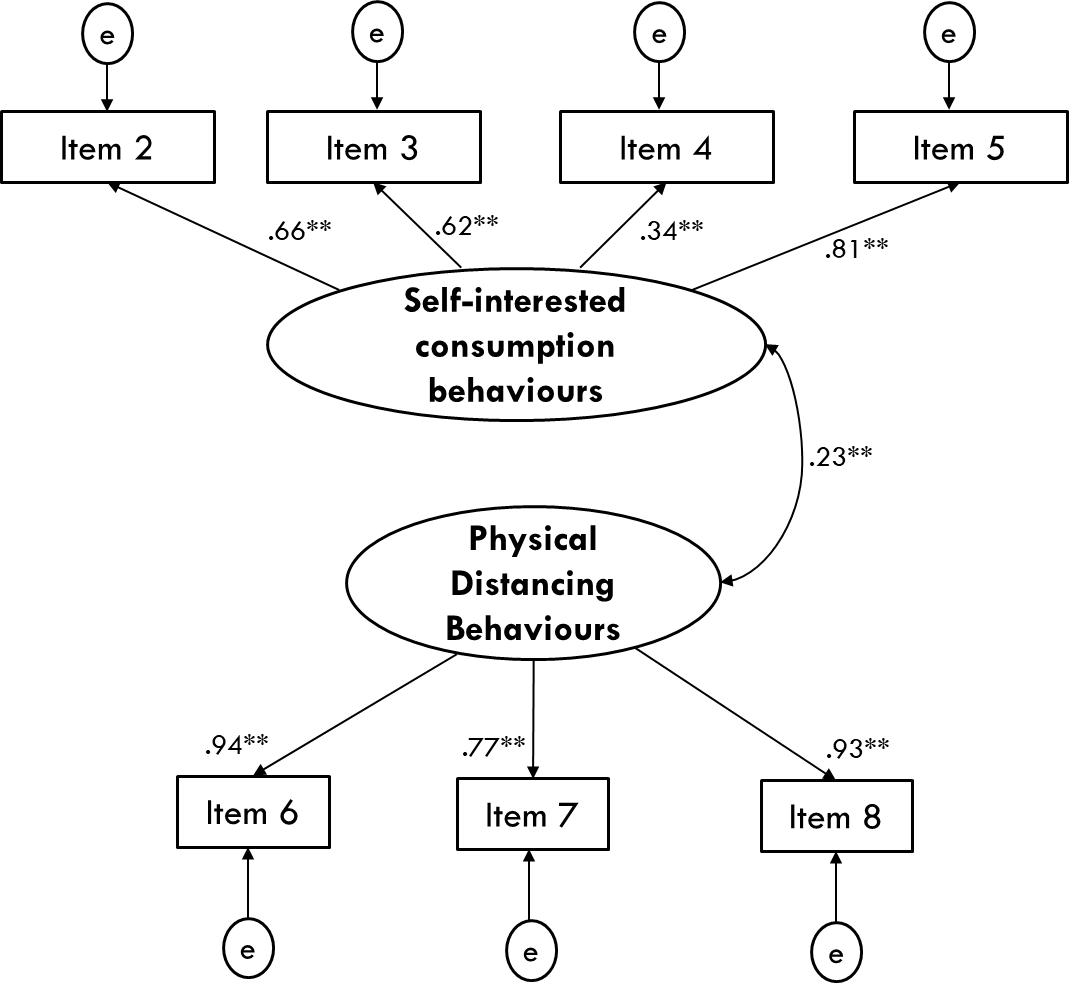

Supplement: S1 Appendix — (DOCX) [file pone.0238682.s001.docx]
